# Development and validation of the Readiness to Train Assessment Tool (RTAT)

**DOI:** 10.1186/s12913-021-06406-3

**Published:** 2021-04-28

**Authors:** Ianita Zlateva, Amanda Schiessl, Nashwa Khalid, Kerry Bamrick, Margaret Flinter

**Affiliations:** Weitzman Institute, Community Health Center, Inc., Middletown, CT USA

**Keywords:** Health center, Organizational readiness, Health professions training, Implementation, Dissemination, Measure, Survey, Development, Reliability, Validity

## Abstract

**Background:**

In recent years, health centers in the United States have embraced the opportunity to train the next generation of health professionals. The uniqueness of the health centers as teaching settings emphasizes the need to determine if health professions training programs align with health center priorities and the nature of any adjustments that would be needed to successfully implement a training program. We sought to address this need by developing and validating a new survey that measures organizational readiness constructs important for the implementation of health professions training programs at health centers where the primary role of the organizations and individuals is healthcare delivery.

**Methods:**

The study incorporated several methodological steps for developing and validating a measure for assessing health center readiness to engage with health professions programs. A conceptual framework was developed based on literature review and later validated by 20 experts in two focus groups. A survey-item pool was generated and mapped to the conceptual framework and further refined and validated by 13 experts in three modified Delphi rounds. The survey items were pilot-tested with 212 health center employees. The final survey structure was derived through exploratory factor analysis. The internal consistency reliability of the scale and subscales was evaluated using Chronbach’s alpha.

**Results:**

The exploratory factor analysis revealed a 41-item, 7-subscale solution for the survey structure, with 72% of total variance explained. Cronbach’s alphas (.79–.97) indicated high internal consistency reliability. The survey measures: readiness to engage, evidence strength and quality of the health professions training program, relative advantage of the program, financial resources, additional resources, implementation team, and implementation plan.

**Conclusions:**

The final survey, the Readiness to Train Assessment Tool (RTAT), is theoretically-based, valid and reliable. It provides an opportunity to evaluate health centers’ readiness to implement health professions programs. When followed with appropriate change strategies, the readiness evaluations could make the implementation of health professions training programs, and their spread across the United States, more efficient and cost-effective. While developed specifically for health centers, the survey may be useful to other healthcare organizations willing to assess their readiness to implement education and training programs.

**Supplementary Information:**

The online version contains supplementary material available at 10.1186/s12913-021-06406-3.

## Background

Health centers are community-based primary health care organizations that provide comprehensive, culturally competent, high-quality services to medically underserved and vulnerable patients in the United States. In 2019, there were nearly 1400 health centers with over 252,000 full-time healthcare providers and staff serving 29.8 million patients [1]. Nevertheless, health centers continue to struggle with workforce recruitment and retention [2]. For example, approximately 13% of the clinical staff positions at health centers across the country remain vacant [3]. Furthermore, the United States is projected to experience a shortage of up to 121,900 physicians by 2032 [1]. In anticipation of this primary care workforce shortage, the Health Resources and Services Administration (HRSA), an agency of the U.S. Department of Health and Human Services, increased funding for health professions training (HPT) and is looking for approaches to solve this issue in a sustainable, long-term manner [4–6].

The creation of an education and workforce pipeline for the health center workforce is one such approach. In recent years, health centers have invested significant effort in the development and implementation of health professions training (HPT) programs [7]. The Teaching Health Center program for training primary care physicians that was authorized under the Affordable Care Act [8], the development of a model of postgraduate nurse practitioner residency and fellowship training led by Community Health Center, Inc. (CHCI) [9], and recent development of federal funding for such programs [10], the expansion of health center based training programs in dentistry and behavioral health [11, 12], and the development of innovative approaches to training medical assistants [13], registered nurses [14], community health workers [15], and others all point to health center interest in this area. In addition to addressing their workforce shortages, health centers’ increased interest in HPT programs can be attributed to several other factors. Among them are the movement towards innovative and integrated clinical and value-based care models, embracing the opportunity to train the next generation of health professionals to a high-performance model of care, and providing them with the tools and skills to address and remove systemic barriers to optimal care for health center patients.

While there is widespread interest in training health professionals across all educational levels and disciplines, many questions and concerns remain regarding capacity, resources, organizational abilities, leadership support and potential impact on the core and extended members of primary care teams and the organization as a whole. It is unclear how ready health centers are to implement or engage with HPT programs. Because the implementation of new programs is consistently contextual and an inherently complex process, the extent to which health center engagement with HPT programs will be successful will differ between health centers. There are many factors that may pose significant challenges to health centers in launching HPT. Specific barriers that health centers face in implementing HPT include limitations in structural capacity to engage with students [16]; finding capable preceptors/supervisors; administrative complexity [17, 18]; and leadership and financial considerations [19, 20].

There is a clear need to determine if HPT programs align with health center priorities and the nature of any adjustments that would be needed to successfully implement and adopt a training program. The extent to which a health center is ready to engage in HPT programs should be assessed by employing a measure of organizational readiness to change or adopt new behaviors. Such evaluation of organizational readiness would allow for early identification and mitigation of many barriers to engage in HPT programs. Furthermore, the potential scale and implications of implementing the programs justify an in-depth analysis of health center readiness, however the relevant literature in this field is scarce. In the change management literature the concept of organizational readiness to change is common [21] and is usually used to describe the extent to which an organization is able and prepared to embrace change and employ innovations [22]. Organizational readiness for change can be viewed as a continuum that starts with the process of exploring a new program and ends with its full implementation [23].

The concept of readiness has been previously examined across various disciplines, as well as within various settings, including hospitals and physician practices [24, 25]. Importantly, research has linked high level of readiness for change to more effective implementation and concluded that organizational readiness to change is a critical antecedent of successful implementation of new programs [24, 26, 27]. However, despite these conclusions assessing readiness to change in healthcare settings is often overlooked [24, 28].

A range of general frameworks and models have been proposed to guide implementation efforts [26, 29–31], though the available literature does not sufficiently address the questions about how to assess health center readiness to engage with and implement an HPT program. In healthcare settings, the main focus has been on the effects of implementing changes in service delivery and care practices. Less is known about what factors determine the successful implementation of education programs or medical curricula changes [32–34] and in particular, what factors influence implementation of HPT programs in health centers.

Measurement of organizational readiness is a field that still remains underdeveloped and fragmented [35], with no gold standard [36]. There are several general measurement instruments described in the readiness for change literature [24, 37], and few valid and reliable tools developed specifically for healthcare settings [28, 38]. Such tools include the Texas Christian University-ORC [39], the ORIC [24], the ARCHO [40], and the instruments specifically developed for undergraduate medical education [41–44]. However, such instruments have been designed to assess readiness for implementation of new policies, guidelines, practices, and changes in curricula rather than HPT programs. Furthermore, the uniqueness of the health centers as teaching settings emphasizes the need for a survey instrument developed to their specific problems, needs and requirements. There is a need to develop an instrument that specifically measures constructs important for the implementation of education and training programs at health centers, where the primary role of the organizations and individuals is healthcare delivery.

To summarize, there is a gap in the literature on how to assess health center readiness to engage with and implement HPT programs. In this study we sought to address this gap by developing and validating a new survey instrument to assess the core dimensions of health center readiness to implement HPT programs.

## Methods

The Weitzman Institute of CHCI conducted the study from July 2018 to June 2019.

Our objectives were to develop and validate a tool to measure and assess health center readiness to engage with and implement HPT programs that is based on organizational readiness theory and experts’ judgement of the most important factors influencing successful HPT program implementation. The tool, named the Readiness to Train Assessment Tool (RTAT), had to be pragmatic and relevant to a wide range of HPT programs and types of health centers regardless of size, scope, location, etc.

To achieve these objectives, we undertook the following methodological steps: 1) development and validation of a conceptual framework; 2) generation of the initial survey item pool; 3) refinement and validation of the survey items; 4) pilot testing of the survey; and 5) psychometric and structural evaluation. To establish pragmatic strength, we followed recommendations from the literature on “pragmatic measurement” [45]. We ensured that RTAT has survey qualities such as relevance to health centers, applicability to a range of settings, feasibility, benchmarking, and actionability.

The study was approved by the CHCI Institutional Review Board Protocol ID: 1133.

### Conceptual framework

During the first phase of the study, we reviewed relevant dissemination and implementation science principles and constructs as well as measures containing individual survey items (surveys, questionnaires, scales, instruments, and tools). Based on this review, we decided to utilize the Organizational Readiness to Change theory (ORC) and the Comprehensive Framework for Implementation Research (CFIR) [26, 31] to guide our work.

For the purposes of the study, we conceptualized the health center workforce needs broadly, at the system level, rather than at the organizational level. Identifying and meeting the needs of the health center workforce at a system level might involve health centers forming partnerships to agree on priorities and actions regarding health professions training. Health centers need to recognize and prioritize their shared goals to meet the workforce needs despite their organizational differences. They need to take a system-wide view of what the workforce needs are and how these needs can be met through HPT implementation.

We broadly defined *‘health professions training’* as any formal organized education or training undertaken for the purposes of gaining knowledge and skills necessary to practice a specific health profession or role in a healthcare setting. Health centers may provide HPT at any educational level (certificate, undergraduate, graduate, professional and/or postgraduate) and in any clinical discipline. For the purposes of this study, the following were considered examples of types of HPT programs:

- Established affiliation agreements with academic institutions to host students or trainees
- Formal agreements with individual students
- Directly sponsoring accredited or accreditation-eligible training programs (across all disciplines and education levels).

Additionally, we defined *‘organizational readiness for change’* as the degree to which health centers are *motivated* and *capable* to engage with and implement HPT programs. We kept this definition more general and in line with the main constructs of the ORC theory (change commitment and change efficacy) [26], mainly because there is no consensus around a definition of organizational readiness for change [36]. Consistent with the ORC theory, while building capacity is a required aspect of successfully getting a health center ready to implement an HPT program, an overall collective motivation, or commitment to engage with HPT is equally and critically important [26, 46].

Since organizational readiness for change is a multi-faceted and multi-leveled construct [26, 47], to be able to measure it, we defined the domains of organizational readiness to implement an HPT program as the broad characteristics or determinants of organizational readiness which the individual survey items should represent. We utilized the CFIR’s constructs [31] as domains to adapt for our survey and include in the study’s conceptual framework.

While organizational readiness is emphasized as important in most implementation frameworks and theories [23, 48], we chose CFIR for two main reasons. First, it brings together numerous theories developed to guide the planning of implementation research. CFIR is a comprehensive framework of 37 constructs within 5 key dimensions (the intervention, the individuals, the inner setting, the outer setting, and the implementation process) that are considered important for the successful implementation of an intervention [31]. Second, we chose the CFIR’s constructs because they can be easily customized to diverse settings and scenarios [36] and thus, have been used in dozens of studies [49, 50]. This was an important consideration, because when creating a survey, it is important to clearly define and communicate the definitions of the domains to be measured to everyone involved in the survey design. Because CFIR provides the constructs’ terms and definitions to adapt and apply for varied implementations [36], we reliably described the domains of organizational readiness to implement HPT programs at health centers in the conceptual framework we developed for the study.

We included the following five domains in the study’s conceptual framework:

(1) characteristics of the HPT program; (2) external context (external to the health center factors that influence implementation); (3) organizational characteristics (health center characteristics such as leadership, culture, and learning climate); (4) characteristics of individuals (actions and behaviors of health center staff); and (5) process (the systems and pathways supporting the implementation of a new HPT program within the health center). See Additional file 1 for the detailed conceptual framework.

During the second phase of the study, this framework was validated by 20 subject matter experts with experience in HPT at health centers. We obtained their opinions through two online focus groups conducted in November, 2018. The experts had to consider every facet of their own implementation experience in order to indicate whether the domains/subdomains of the proposed conceptual framework were directly related to factors facilitating or hindering implementation of HPT programs. Experts’ comments were also invited on the practical utility of measuring each of the domains/subdomains before and during an implementation of a new HPT program. Based on the experts’ recommendations, we eliminated one of the subdomains in the initially proposed conceptual framework (Peer Pressure). The validated framework was in line with the CFIR’s conceptualizations [31].

### Survey item pool

Our initial survey item pool consisted of 306 items found through review of existing surveys in the organizational readiness literature and deemed as relevant [24, 39, 42–44, 51–56]. There was evidence of overall reliability and validity provided for most of these surveys. Although data on the psychometric properties remain to be published for three of the surveys [52–54], they contain items broadly similar in content and wording to items from already validated instruments.

We reviewed all items and deleted the redundant ones. Since the items had to reflect as much as possible the construct to be measured [57] and to fit the purposes of this study, we reworded the remaining items while mapping them to a relevant domain and subdomain in the conceptual framework. During the rewording process, additional changes were intentionally made so that the survey items can be applied to any HPT program and any health center. Rewording and mapping decisions were made by a consensus decision-making process among the members of the research team. After finishing this process, 182 items remained in the item pool for possible inclusion in the survey. This was in line with recommendations for developing an initial item pool that is two to five times greater than the final measure [58, 59] which provides a sufficient margin for selecting the best and most robust item combination in the final version of the survey.

### Content validation

During the third phase of the study, we used a modified, web-based, Delphi procedure to further refine and validate the survey item pool. The Delphi approach is a well-established research method to reach consensus on a specific subject matter among a panel of experts through feedback of information in several, usually 2–4 rounds [60–62].

Thirteen subject matter experts were recruited and agreed to serve as panelists in all three Delphi rounds in February–April 2019. For each Delphi round, the experts received an email with instructions and a link to the online survey which was administered via Qualtrics Research Core software.^1^

The Delphi panelists assessed each survey item based on their level of agreement with the item’s *appropriateness* and *ability to measure* the relevant domain/subdomain in the conceptual framework (rounds 1 and 2; a 5-point Likert scale) and the item’s *importance* for organizational readiness to engage with HPT programs (round 3; 9-point Likert scale). In addition, the panelists had the opportunity to suggest rewording of items as well as new items for the survey. For each round, they had at least 1 week to review and assess the items and to propose changes. The Delphi procedure was closed after round three. At this time, the experts were invited to provide their feedback regarding the design of the final survey.

After each round, all experts received an individual report with their own scores for each item compared against the average group scores. After reviewing the reports, the experts had the opportunity to change and re-submit scores. Based on the level of agreement reached among the panelists in that round, items were either accepted in the survey, eliminated, modified or added back for re-evaluation. Only survey items reaching the required level of consensus by more than 80% of the panelists were retained in the final survey. In rounds 1 and 2, these levels were the highest two points of the ‘appropriateness’ and “ability to measure’ scales. In round 3, only items scoring as “critically important” (highest 3 points) were retained.

Over the three Delphi rounds, there was a reduction in the number of survey items and subdomains. The final content-validated survey comprised 65 survey items (statements) over 5 domains and 22 subdomains.

### Pilot testing of the survey

During the final phase of the study, we pilot-tested the survey with staff from health centers across the United States. We used the collected responses in the psychometric and structural validation of the survey.

In the instructions, the pilot test participants were asked to answer questions related to their health center’s overall readiness and future plans to engage with HPT programs. They had to indicate the extent to which they agree with the 65 survey statements as they pertain to their health center’s readiness to engage with HPT program(s). If the health center was considering more than one HPT program, for the purposes of these questions, participants were encouraged to think about only one of them and to respond openly and honestly, based only on their own judgment, regardless of what others expect or what is socially acceptable at their health center.

A five-point Likert scale was chosen for the 65 survey statements because it approximates an interval-level measurement and has been shown to create the necessary variance to examine the relationships among survey items and to create acceptable internal consistency reliability estimates [63]. Items were positively stated with one exception; the negatively stated item was reverse coded during the analysis but eliminated during the statistical testing of the survey. The distributed questionnaire also contained questions to collect data on the demographic and professional characteristics of the individual respondents and the characteristics of their health centers (e.g., number of patients served, HPT efforts). Additional questions were also added for testing the convergent, discriminant, and predictive validities of the survey.

Before the distribution of the questionnaire, the survey items and the instructions to the respondents were uploaded into Qualtrics and field-tested for ease of comprehension by two staff members at CHCI/Weitzman Institute who also tested and confirmed the question routing and response choices. Their suggestions contributed to the final version of the distributed questionnaire.

To recruit pilot-test participants, we used both convenience and purposive sampling methods and leveraged the CHCI network. An email invitation with a link to the survey was distributed in four waves to 9209 potentially eligible individuals. In addition, three email reminders were sent over the period May–June 2019.

We screened respondents for eligibility for the survey. To be included in the study, respondents had to be current employees of a health center in the United States. They also had to feel sufficiently informed to answer questions related to current or future engagement with HPT program(s) at their health center. Respondents were provided study information and consented by proceeding to complete the survey. The range for completing the survey was between 15 to 25 min.

Of all 386 respondents, 301 (78%) screened as eligible for participation and engaged with the survey. A final sample of 212 health center employees who completed all survey items were retained for psychometric and structural validation. The pilot test sample that was included in the analysis represented a wide range of demographic and professional characteristics, as well as roles and experience with HPT programs. Seventy-one percent of the participants were female and 61.5% identified as White/Non-Hispanic. Forty-nine percent had graduate degree, while 27.7% identified as having a clinical doctorate. The respondents’ health centers were located in 41 US states and had different characteristics and levels of HPT engagement. More details about the sample characteristics can be seen in Table 1, while the characteristics of the health centers of the participants are presented in Table 2. In addition, Table 3 shows the states where the health centers are located.

**Table 1:** Pilot-test respondent characteristics

N (%)		
**Age** (*N* = 200)	< 30	3 (1.5)
	31–40	37 (18.5)
	41–50	44 (22.0)
	51–60	53 (26.5)
	61–70	48 (24.0)
	Over 70	8 (4.0)
	Prefer not to answer	7 (3.5)
**Gender** (*N* = 200)	Female	142 (71)
	Male	58 (29)
**Racial/ethnic group** (*N* = 200)	Black/African American	27 (13.5)
	Hispanic/Latino	13 (6.5)
	White/Non-Hispanic	123 (61.5)
	Other	37 (18.5)
**Highest level of education completed**^a^ (*N* = 202)	Undergraduate	47 (23.3)
	Graduate	99 (49.0)
	Clinical Doctorate	56 (27.7)
**Involvement in health center’s health professions training program(s)**^a^ (*N* = 202)	Leadership	136 (67.3)
	Management/Operations	96 (47.5)
	Administration	103 (51.0)
	Educator	71 (35.1)
	Preceptor/Mentor	112 (55.4)
	Not Applicable	60 (29.7)
**Years of experience with health professions training program(s)** (*N* = 200)	No experience	12 (6)
	Less than 1	44 (22)
	1–5	36 (18)
	6–10	15 (7.5)
	More than 10 years	93 (46.5)
**Current role at health center** ^a^ (*N* = 201)	Academic	19 (9.5)
	Clinical	93 (46.3)
	Administration	157 (78.1)
	Education	30 (14.9)
	Policy	21 (10.4)
	Government	1 (0.5)
	Other	12 (5.8)
**Number of years working at current health center** (*N* = 200)	0–5	77 (38.5)
	6–10	36 (18)
	More than 10 years	87 (43.5)

^a^ Respondents were allowed to select multiple options for this question

**Table 2:** **Characteristics of the health centers of the pilot test participants**

**Health center’s overall stage of engagement with health professions training programs** ^a^ (*N* = 201)	**N**	**(%)**
Early stage- awareness of health professions training programs	44	21.9
Planning for implementation of first HPT program	18	9.0
Implementation of first HPT program	16	8.0
Sustaining first HPT program	38	18.9
Expansion of additional HPT programs	85	42.3
**Type(s) of health professions training programs at health center** ^a^ (*N* = 211)	**N**	**(%)**
Not engaged in health professions training program(s)	11	5.2
Informal training (e.g., shadowing, rotations, experiential training)	135	64.0
Established affiliation agreements with academic institutions to host students	162	76.8
Formal agreements with individual students	100	47.4
Directly sponsoring accredited or accreditation eligible training programs (across all disciplines and education levels)	86	40.7
Other	17	8.1
**Past, current, or future health professions training efforts at the health center are at the following educational level(s)** ^**a**^ (*N* = 211)	**N**	**(%)**
Certificate	113	53.6
Paraprofessional	65	30.8
Undergraduate	95	45.0
Graduate	161	76.3
Post-Professional	112	53.1
**Past, current, or future health professions training efforts at the health center are in the following clinical discipline(s)** ^**a**^ (*N* = 212)	**N**	**(%)**
Medical (MD, NP, PA, CNM, MA, Pharmacist)	180	84.9
Nursing (RN, LPN)	129	60.8
Dental (Dentist, Dental Hygienist, Dental Assistant)	109	51.4
Behavioral Health (Psychiatry, Psychiatric NP, PsyD, MA-Level Therapist)	138	65.1
Other	44	20.7
**Approximate number of students/trainees trained per year at the health center** (*N* = 201)	**N**	**(%)**
0–25	132	65.7
26–50	28	13.9
51–100	22	10.9
> 100	19	9.5
**Number of unduplicated patients in the most recent 12-month period** (*N* = 200)	**N**	**(%)**
Under 25,000	93	46.5
25,000-99,999	76	38
100,000-200,000	24	12
Over 200,000	7	3.5
**Health center’s current certification status for Patient Centered Medical Home** (*N* = 200)	**N**	**(%)**
Level I	18	9
Level II	28	14
Level III	119	59.5
Not certified	35	17.5
**Site(s) of the health professions training program designated as rural** (*N* = 194)	**N**	**(%)**
Yes	83	42.8
No	101	52.1
Uncertain	10	5.2

^a^ Respondents were allowed to select multiple options for this question

**Table 3:** Pilot test respondents: state where health center is located

State where health center is located	N (respondents)	(%)
Alabama	3	1.5
Arizona	12	6
Arkansas	2	1
California	24	12
Colorado	6	3
Connecticut	16	8
Delaware	1	0.5
Florida	5	2.5
Georgia	4	2
Hawaii	10	5
Idaho	1	0.5
Illinois	1	0.5
Indiana	4	2
Iowa	1	0.5
Kansas	1	0.5
Kentucky	1	0.5
Louisiana	2	1
Maine	3	1.5
Maryland	1	0.5
Massachusetts	11	5.5
Michigan	7	3.5
Minnesota	1	0.5
Mississippi	3	1.5
Missouri	4	2
Montana	3	1.5
New Hampshire	2	1
New Jersey	3	1.5
New Mexico	3	1.5
New York	14	7
North Carolina	7	3.5
Ohio	4	2
Oklahoma	1	0.5
Oregon	2	1
Pennsylvania	1	0.5
South Carolina	6	3
Tennessee	4	2
Texas	10	5
Virginia	1	0.5
Washington	9	4.5
West Virginia	1	0.5
Wisconsin	2	1
US Territories	3	1.5
**Total**	**200**	

### Psychometric and structural validation

Exploratory factor analysis (EFA) was used to explore the internal structure and validity of the survey [64, 65]. By using this method, the survey items are being reduced, but the survey still provides nearly the same amount of information as the original number of items. First-order EFA was carried out by means of principal axis factoring and rotated using the promax procedure with Kaiser’s Normalization to an oblique solution to generate a factor solution for the survey. To assess compliance with the distribution requirements, Bartlett’s test of sphericity and the Kaiser-Meyer-Olkin (KMO) measure of sampling adequacy were used. In order to estimate the number of significant item factors, Kaiser’s criterion [66] and Cattell’s scree-plot [67] were used. Cronbach’s alpha coefficients, as well as the average correlations between the items of each factor were calculated to examine the internal consistency reliability and unidimensionality of the retained factors of the survey [68, 69]. Convergent, discriminant, and predictive validity were also examined.

Data were analyzed using IBM SPSS Statistics 20 Software Package [70].

## Results

An exploratory factor analysis was used to reduce the set of survey items to a more parsimonious set and to identify those items that would load as predicted. The principal-components method of analysis was used as it is the most common factoring method and because it accounts for common, specific and random error variances [71, 72].

To determine the suitability of the data for factor analysis, the sample size and the relationship between the responses to the items were examined. The sample size was deemed adequate to appropriately conduct the analyses. The number of subjects with usable data (212) was larger than three times the number of variables (65). In most cases, as long as item intercorrelations are reasonably strong, a sample size of 150 cases is sufficient to produce an accurate solution in exploratory factor analysis [73].

The intercorrelation matrix revealed that underlying structures do exist. Both Bartlett’s test of sphericity (11,198.358; *p* < .001) and the KMO measure of sample adequacy (.934) confirmed that the properties of the correlation matrix of the item scores were suitable for factor analysis, in terms of the guidelines recommended by Hair et al., 2014 [74]. In the first round of EFA, the responses on the 65 items of the survey were inter-correlated and rotated by means of the promax procedure to an oblique solution. The eigenvalues and the scree plot obtained from this initial extraction were examined, which provided a preliminary indication of the number of factors represented by the data. Based on Kaiser’s [66] criterion (eigenvalues greater than 1.0), 11 factors were extracted. The 11 factors explained 70.8% of the variance in the factor space of the data.

Next, the items included in the extracted 11 factors were scrutinized. All the items which had factor loadings <.50 or cross-loadings < 0.2 were removed. To ensure that each factor was well measured, factors with less than 3 items were also removed.

Only 41 items loading on the first 7 factors were retained and they were subjected to a second round of EFA with promax rotation. The KMO measure of sample adequacy (.943) and the significant sphericity (7299.149, *p* < .001) of the data indicated that the properties of the correlation matrices of the 41 item scores were likely to factor well. The expected seven factors with eigenvalues greater than one were extracted. Inspection of the eigenvalues and the scree-plot confirmed that seven factors had been properly determined. These factors explained 72.64% of the total variance in the data. The communality of all the items was .50 or more; the average communality of all items was .72. This extraction together with the study’s conceptual framework was used to determine the ultimate number of underlying factors and items to be retained. The factor loadings for the survey items in the finalized factor structure are provided in Table 4.

**Table 4:** Factor loadings for the survey items in the finalized factor structure

Survey Item	Subscale (component)						
	Implementation Plan	Readiness to Engage	Implementation Team	Financial Resources	Evidence Strength & Quality of the HPT Program	Relative Advantage of the HPT Program	Additional Resources
The implementation plan for this program... **- identifies strategies for unanticipated obstacles.**	**.937**						
The implementation plan for this program... **- includes a communication plan to share progress with multiple stakeholders, regardless of their direct involvement (e.g., communication to the funder, board of directors, leadership, staff, patients, community partners).**	**.935**						
The implementation plan for this program... **- intends to compare anticipated vs. actual progress.**	**.911**						
The implementation plan for this program... **- allows for adequate time to reflect on and evaluate whether implementation team members are satisfied with the progress outcomes.**	**.881**						
The implementation plan for this program... **- identifies strategies for gaining staff confidence (e.g., employees feel safe and have accepted the new changes).**	**.866**						
The implementation plan for this program... **- includes an evaluation plan conducted with both quantitative and qualitative measurements.**	**.863**						
The implementation plan for this program... **- includes tracking of the implementation progress (e.g., milestones, spending).**	**.857**						
The implementation plan for this program... **- allows for adequate time to reflect on and evaluate factors influencing the program’s success.**	**.855**						
The program will be carried out or accomplished according to an implementation plan that... **- is being updated and shared with leadership and staff on a regular basis.**	**.853**						
The implementation plan for this program... **- identifies a strategy to monitor the impact of the health professions training program on productivity.**	**.811**						
The implementation plan for this program... **- intends to use data to inform program delivery and to monitor fidelity to the program’s model.**	**.777**						
The program will be carried out or accomplished according to an implementation plan that... **- allows for staff input and opinions.**	**.762**						
The program will be carried out or accomplished according to an implementation plan that... **- has well-formulated and sufficiently detailed action steps and timelines to guide engaged staff.**	**.642**						
The program will be carried out or accomplished according to an implementation plan that... **- can be modified or revised due to unexpected barriers.**	**.552**						
The implementation plan for this program... **- ensures the necessary resources to implement the program are available.**	**.539**						
At our health center: **Engaging with health professions training is compatible with our organizational culture.**		**1.005**					
**Leaders and managers have taken steps to encourage staff to engage with health professions training.**		**.908**					
At our health center: **Engaging with health professions training is feasible and appropriate in the life of the organization at this time.**		**.900**					
At our health center: **Collaboration is encouraged.**		**.870**					
At our health center: **Engaging with health professions training is a high priority.**		**.867**					
**Staff are well-informed about the progress of existing and/or planned health professions training programs.**		**.851**					
At our health center: **Our mission, vision and values towards health professions training are well-communicated and shared.**		**.635**					
**Our health center is able to leverage our external relationships to support health professions training program implementation.**		**.576**					
The implementation team members for this health professions training program … **have release (protected) time for this health professions training program.**			**.828**				
The implementation team members for this health professions training program … **can keep the momentum going in implementing this health professions training program (e.g., in the face of challenges, over the long run).**			**.784**				
**There is a comprehensive implementation team in place for the program (e.g., representatives from multiple departments of the organization, a champion for the program).**			**.735**				
The implementation team members for this health professions training program … **have clearly defined roles and responsibilities.**			**.723**				
**Our health center is financially able to trial health professions training programs.**				**.974**			
**The following resources are available and sufficient to implement and carry out the health professions training program: - Financial, including costs related to implementation.**				**.693**			
**Regarding health professions training, our health center has an overall budget that accounts for our mission to act as a teaching organization.**				**.643**			
Our health center has enough evidence to support that: **The health professions training program is flexible enough to be redesigned to meet the needs of our health center.**					**.896**		
Our health center has enough evidence to support that: **If the organization engages with this health professions training program, it will be beneficial for our health center workforce.**					**.895**		
Our health center has enough evidence to support that: **The program will help trainees better adapt to relevant best practices at health centers.**					**.812**		
Our health center has enough evidence to support that: **The health professions training program has clear structure and goals.**					**.605**		
The majority of staff members feel that the program will: **- Lead to better recruitment of health professionals (e.g., the pool of qualified applicants will increase).**						**.878**	
The majority of staff members feel that the program will: **- Better meet anticipated workforce needs.**						**.846**	
The majority of staff members feel that the program will: **- Lead to improved patient care outcomes.**						**.742**	
The majority of staff members feel that: **The costs in time and resources required to implement this health professions training program are worth the potential benefit.**						**.530**	
The following resources are available and sufficient to implement and carry out the health professions training program: **- Staff (e.g., interested and qualified preceptors/supervisors).**							**.902**
The following resources are available and sufficient to implement and carry out the health professions training program: **- Evaluation resources.**							**.564**
The following resources are available and sufficient to implement and carry out the health professions training program: **- Assistance for staff (e.g. tools, training, coaching, and ongoing support as they adjust to the changes due to implementation of the health professions training program).**							**.562**

Extraction Method: Principal Component Analysis

Rotation Method: Promax with Kaiser Normalization

Rotation converged in 10 iterations

The survey items that were eliminated after factor analysis are presented in Additional file 2. The bivariate Pearson correlations of all items were calculated to provide an overview of their relations and to check for multicollinearity. No multicollinearity was detected; there were no correlation coefficients of .8 or higher.

Thus, in order to measure health center readiness to engage with and implement HPT programs, a new 41-item scale for health center readiness was developed with seven subscales corresponding to the seven factors identified through factor analysis. The subscale variables were created by taking the average of the survey items belonging to a subscale (component) as shown by the factor analyses.

The new scale was constructed as a reflective measure. Reflective measures are expected to have high inter-intercorrelations and to “reflect” the latent construct, in this case - the health center readiness to engage with and implement HPT programs. Cronbach’s alpha for the overall scale was excellent (.969). The high reliability of the overall scale indicates strong item covariance or homogeneity, meaning that the survey items measure the same construct well [75]. Further reliability analysis showed that the factors or subscales also had good to excellent internal consistency (.787 to .970).

The seven subscales that emerged from the data analysis represent seven areas of readiness: readiness to engage (8 items), evidence strength and quality of the HPT program (4 items), relative advantage of the HPT program (4 items), financial resources (3 items), additional resources (3 items), implementation team (4 items), and implementation plan (15 items). See Table 5 for details about the subscales.

**Table 5:** Descriptions, number of survey items, and Cronbach’s alphas of the seven subscales ^a^

Subscale (factor)	Brief Description of the Subscale	Number of Items	Cronbach’s alpha
**Readiness to Engage**	Indicators of the health center’s overall readiness and commitment to engage with health professions training.	8	.905
**Evidence Strength & Quality of the HPT Program**	Stakeholders’ perceptions of the quality and validity of evidence supporting the belief that the HPT program will have desired outcomes at their health center.	4	.899
**Relative Advantage of the HPT program**	Stakeholders’ perceptions of the advantage of engaging with/implementing the HPT program versus an alternative solution.	4	.832
**Financial Resources**	The level of financial resources dedicated for implementation and ongoing operations	3	.787
**Additional Resources**	The level of additional resources dedicated for implementation and on-going operations, including appropriate staff and assistance for staff (e.g. evaluation resources, tools, training, and coaching).	3	.840
**Implementation Team**	This subscale is about the individuals involved with the HPT implementation process who can formally or informally influence this process through their knowledge, attitudes, and behaviors. They are effective in overcoming indifference or resistance that the implementation of an HPT program may provoke in the health center.	4	.913
**Implementation Plan**	This subscale is associated with the implementation process. Successful engagement usually requires an active change process aimed to achieve effective implementation of the HPT program(s). The subscale measures the degree to which a scheme or method of behavior and tasks for implementing an HPT program are developed in advance, and the quality of those schemes or methods.	15	.970

^**a**^ The subscales and their descriptions are in line with the conceptualizations in the study’s conceptual framework (adapted from the Consolidated Framework for Implementation Research – [31])

During the reliability analysis none of the survey items were deleted, because the Cronbach’s alphas were already higher than .80 for six of the subscales and the different survey items contributed to the stability and completeness of the subscales they belonged to. The analyses for alphas if items were deleted did not show significant improvements.

Within the overall readiness scale, health centers can evaluate their readiness for a specific HPT program using these seven subscales. The survey allows for three levels of assessment and scoring: at the survey item, subscale, and overall scale levels by obtaining their mean (average) scores. Each survey item can and should have its response analyzed and reviewed separately.

Mean (average) scores may range anywhere from 1 to 5 with 5 indicating highest readiness to engage with and implement a specific program. To ease interpretation, these means can then be used to assign one of three levels of readiness: *developing readiness* (mean scores 1.0–2.9); *approaching readiness* (mean scores 3.0–3.9); and *full readiness* (mean scores 4.0–5.0) - for each survey item, subscale, and for the overall scale.

Further, we confirmed by sizeable correlations with other measures the convergent validity of RTAT. RTAT was expected to correlate with two questions: the first one, rating the health center’s overall readiness to engage with HPT (agreement, on a 1–5 scale); and the second one, rating the health center’s readiness to engage in quality improvement activities (from poor = 1 to excellent = 5). We tested the discriminant validity of RTAT by determining no relationships existed with respondents’ gender and ethnicity, as concepts unrelated to the organizational readiness of the health center. Lastly, we tested and confirmed the predictive validity of RTAT with a question rating the overall quality of the HPT provided at the employee’s health center (from poor = 1 to excellent = 5).

## Discussion

In this paper, we presented the process we used to develop and validate a survey to measure health center readiness to engage with and implement HPT programs. We used survey development methods that are closely aligned with current recommendations and included the involvement of subject matter experts in the development and validation process; the use of consensus methods and theory to develop the survey items; field-testing; and pilot-testing of the developed survey. The result of this stepwise process, the Readiness to Train Assessment Tool (RTAT), is a theoretically-based, valid and reliable 41-item measure with 7 subscales. It was designed to assess readiness from a health center staff perspective and to be applicable to a wide range of HPT programs and types of health centers.

RTAT addresses the difficult task of measuring health center readiness in a field with few valid assessment tools. However, similar to other measures that are based on CFIR, the survey subscales assess factors such as perceived complexity, relative advantage, and knowledge and beliefs about the intervention being implemented; available resources; implementation climate, implementation team, and implementation plan [76].

RTAT’s seven subscales or core elements suggest that successful implementation is dependent on the health center being receptive to the change, there is an agreement among staff members that the evidence about the HPT is robust, there are efficient and effective implementation processes in place, and adequate resources are available for implementation, including dedicated staff, money, and time. Since organizational readiness involves collective action from many staff members, the factors reflected in the subscales are illustrative of the collaborative nature of the HPT engagement and implementation work. The survey items outline the multiple steps and considerations of an implementation process that is closely aligned with the context of a health center. As an example, many implementation team members are also providing patient care and need to have release (protected) time for HPT program implementation.

Although specific HPT programs and health centers may require very specific organizational changes, there is a range of key requirements or determinants of implementation success which remain similar across the HPT programs and across all health centers. Throughout the development and validation process, our focus was on these common key requirements to ensure the generalizability and applicability of the survey to various health centers and HPT programs. For these reasons, we performed the survey’s psychometric evaluation with data collected in diverse contexts (health centers in 41 US states with various levels of HPT engagement), from staff with various demographic and professional characteristics (roles/experience with HPT programs), and from the standpoint of different HPT programs. We believe this variability is one of the strengths of our development and validation process. However, we also need to explore the possibility that different groups of employees respond differently to RTAT questions. The extent of different perceptions relating to organizational readiness, any underlying reasons for such differences (e.g., demographic and professional characteristics, geographic location, and different access to information), and their impact on the results from the survey should be examined as a next step.

Furthermore, the pilot test data showed that our respondents were from health centers at different stages of implementation of HPT programs, which could have an impact on the quality of respondents’ responses. In our study, we believe that having responses from health centers that are at different stages of implementation adds needed variability to the data. When creating a scale, considerable variability is desirable because it ensures the ability of items in the scale to measure the variability of the phenomena [57]. Lack of variability makes the scaling efforts challenging and if an item lacks variability it is usually deleted during the analysis.

However, there is recent research that suggests that there are important differences in the relative significance of the broader readiness components (motivation, general capacity, and intervention-specific capacity) at different stages of implementation [77]. While there should be more research into this problem, there are some indications that different readiness issues should be considered at different stages of implementation.

It should also be noted that because of our strict inclusion criteria, the respondents in our pilot test sample might overrepresent health center employees who are interested in HPT program implementation thus limiting generalizability in this context. However, respondents represented all geographic areas in the United States, had a variety of roles and probably were more actively involved in the implementation of HPT programs at their health centers. We believe that this specific group of respondents was better placed to accurately report on the health centers’ readiness to engage with HPT programs and the specific factors measured by the survey given their sufficient knowledge of the health centers’ plans adding credibility to their perceptions. The exclusion of employees who did not feel sufficiently informed about their organization’s current or future engagement with HPT might have influenced the data and the results. However, we think this was necessary and important limitation of the study to ensure that the survey instrument is built by using the most reliable data.

As a survey instrument, RTAT demonstrates both psychometric [45, 78, 79] and pragmatic [45] strength. In establishing RTAT’s validity, we considered two important aspects: the survey measure is theory-based and its variance reflects the variance in the actual construct being measured [80]. We used exploratory factor analysis which is the most commonly used statistical method to establish construct validity [64, 65]. We reported 72% of total variance explained by the final EFA model; excellent Cronbach’s alpha of .97 for the overall scale, and Cronbach’s alphas of at least .79 for the seven subscales.

Although we attempted to achieve high standards with respect to the psychometric properties of the RTAT, we believe that additional work assessing the RTAT dimensionality should be considered. Confirmatory factor analysis (CFA) should be used in a larger study to further verify the adequacy of the conceptual structure of the instrument (the scale/subscales determined through the EFA) and to investigate whether the instrument has the same structure across different groups/ samples. In our study, while the size of the sample was adequate to meet the requirements for EFA, it was not large enough to allow the simultaneous cross-validation of the instrument with CFA, to assess the extent to which the factors proposed by the EFA fit the data and to confirm the stability of the structure.

Additionally, we acknowledge that not having a specific construct on general capacity in the final survey structure might be a potential limitation of the instrument. Our study had an exploratory approach and no clear formal hypothesis on the expected dimensionality or factor structure for the instrument was produced based on theory. CFIR provided a general idea about what to expect and key information for selecting the initial set of items, but we had no clear predetermined structure in mind for our instrument and did not seek to confirm the survey structure based on the CFIR’s domains. Rather, we used expert opinion and factor analytic procedures to explore all possible interrelationships between the set of items and were open to accept the grouping of items that presented the best structure that was both theoretically and statistically sound. Based on this methodology, general capacity items did not receive much support in the final structure of our instrument. While the initial set of possible survey items included items on general capacity, most of these items were eliminated during the Delphi procedure by our subject matter experts.

As a pragmatic measure, RTAT is expected to have a low burden of administration and to provide maximum value for the efforts for administering it. Its use for benchmarking would allow for measuring the impact of any changes after the start of the program [39], therefore, it can be used during the initiation and the implementation phases [81].

Additionally, since implementation is a complex process that requires the joint and coordinated efforts of the organization as a whole [82], RTAT was designed to measure readiness for change at the individual health center level. Therefore, the wording of the survey items and the instructions to the survey respondents were critical to ensure that all items can be answered by all health center employees; are relevant to all implementation staff roles; and are applicable at any stage of implementation of any HPT program. Additionally, the Likert-type rating scales we chose for the survey statements, allow for answers that are not compromised by forced completion. Since the performance of a scale, in general, is not affected by the approach used to calculate the scores (weighted vs unweighted) [59], we decided to use the unweighted scoring (means). Therefore, each individual survey item contributes equally to the subscale and overall scale score.

In defining the domains and developing the items to measure the domains, we followed a “best practice” approach [59] that combines two types of methods, deductive and inductive [83] . The use of the deductive method involved exploring the literature for existing theories and measures in order to define organizational readiness for change and to identify relevant domains and survey items. We extended the CFIR and organizational readiness conceptualizations into survey items by judging and mapping all items against domain and subdomain definitions. This ensured that RTAT is linked and consistent with a larger theoretical framework which is one of the strengths of our development process.

The use of the inductive method involved conducting focus groups and Delphi rounds with subject matter experts. Their contributions to the development and validation of the survey ensured its strength to measure the most important and relevant to the health centers organizational readiness determinants. By using both deductive and inductive methods (both literature review and expert opinion) we established not only the theoretical underpinning but also the best possible face and content validity for RTAT. Furthermore, we hope that because of its strong face and content validity health centers will view the survey as appropriate and acceptable for their use [79].

RTAT’s seven subscales identify critical components of organizational readiness that health centers need to address as soon as they start considering engagement with a specific HPT program. Implementation is a complex and multi-factorial process; therefore, it is important to have a better understanding of the influencing factors and mediators of successful implementation, especially during the early planning stages. RTAT will not only help health centers better understand these critical components but will also allow for early identification and mitigation of barriers for engagement. Based on the information from the survey, health centers will be able to not only identify specific issues and areas where additional focus may be needed, but to also select the most relevant and effective strategies for implementation. Implementation strategies are considered essential tools for supporting the implementation process [84] and should be linked to specific determinants of implementation success. There are several lists and taxonomies, including the Expert Recommendations for Implementing Change (ERIC) [85] and the Behavior Change Technique [86], that can be used as a starting point for developing and selecting strategies based on the RTAT results. The developed strategies should be incorporated accordingly into the implementation of HPT programs nationwide.

## Conclusions

In conclusion, we developed a parsimonious and practical survey that assesses health center readiness to engage with and implement HPT programs as it is perceived by the health center employees. The final survey, the Readiness to Train Assessment Tool, is a 41-item, 7-subscale, organizational readiness scale that is both valid and reliable. It represents determinants of readiness judged as ‘critical to evaluate’ by subject matter experts. The survey measures domains such as readiness to engage, evidence, strength and quality of the HPT program, relative advantage of the HPT program, financial resources, additional resources, implementation team, and implementation plan. The advantage of the RTAT is that it covers organizational readiness dimensions that are relevant to a wide range of HPT programs and types of health centers. While developed specifically for health centers, the survey may be useful to other healthcare organizations willing to assess their readiness to implement an HPT program.

Most importantly, the RTAT meets a significant need at the national level. As a next step, it provides an opportunity to formally assess the readiness of all health centers in the United States to train all types of health professions at any education/training level and across all disciplines [87]. RTAT results from this assessment can address concerns regarding capacity, resources, and organizational abilities when launching any HPT program(s). This assessment can inform HRSA on policies, programs, and funding. It can also assist in the continued collaboration between National Training and Technical Assistance Partners and other HRSA partners (e.g. Primary Care Associations and Health Center Controlled Networks) by informing the development of targeted workforce technical and training assistance for the nation’s health centers [87]. Thus, when followed with appropriate change strategies, the readiness assessments could make the engagement and implementation of HPT programs, and their spread across the United States, more efficient and cost-effective.

Because readiness is a critical predictor of implementation success and because a validated measure of health center readiness to implement HPT programs is needed for both research and practice, the newly developed measure has the potential for high impact.

## Data Availability

The datasets used and analyzed during the current study are available from the corresponding author on reasonable request.

## files

**Additional file 1.** Study’s conceptual framework. **Additional file 2.** Survey items that were eliminated after factor analysis.

## Abbreviations

HPT: Health Professions Training
RTAT: Readiness to Train Assessment Tool
HRSA: Health Resources and Services Administration
CHCI: Community Health Center, Inc.
ORC: Organizational Readiness to Change
CFIR: Comprehensive Framework for Implementation Research
EFA: Exploratory factor analysis
KMO: Kaiser-Meyer-Olkin

## Footnotes

1 Copyright© 2019 Qualtrics. Qualtrics and all other Qualtrics product or service names are registered trademarks or trademarks of Qualtrics, Provo, UT, USA. https://www.qualtrics.com
