# Supplementary material for: Development and validation of the Readiness to Train Assessment Tool (RTAT)
Source: BMC Health Serv Res. 2021 Apr 28;21:396. doi: 10.1186/s12913-021-06406-3 (PMC8082650; doi:10.1186/s12913-021-06406-3)
Supplement: Supplementary file 1 — Additional file 1. Study’s conceptual framework. [file 12913_2021_6406_MOESM1_ESM.docx]

# **Readiness to Engage with Health Professions Training (HPT) Programs: Study Conceptual Framework**

| **Readiness to Engage with Health Professions Training (HPT) Programs**  **Study Conceptual Framework***^a^* |
| --- |

| **Construct** | | **Short Description**  *(Factors that may influence engagement/implementation* *of HPT programs from the perspective of the collective as opposed to the individual).* |
| --- | --- | --- |
| **I. CHARACTERISTICS OF THE HEALTH PROFESSIONS TRAINING PROGRAM** | | The first major domain is related to characteristics of the HPT program(s) being implemented into a particular health center. HPT programs are often complex, multifaceted, and have many interacting components. |
| A | Program Source | Perception of key stakeholders about whether the HPT program is externally or internally developed. An HPT program may be internally developed as a solution to a problem, or may be developed by an external entity. The legitimacy of the source may also influence engagement/implementation. |
| B | Evidence Strength & Quality | Stakeholders’ perceptions of the quality and validity of evidence supporting the belief that the HPT program will have desired outcomes. |
| C | Relative Advantage | Stakeholders’ perception of the advantage of engaging with/implementing the HPT program versus an alternative solution. |
| D | Adaptability | The degree to which an HPT program can be adapted, tailored, refined, or reinvented to meet local needs. |
| E | Trialability | The ability to test the program on a small scale in the organization, and to be able to reverse course (undo implementation) if warranted. |
| F | Complexity | Perceived difficulty of implementation of the HPT program. Complex programs require significant reorientation and non-routine processes that produce significant changes in the organization’s activities. The complexity of the program is reflected by the duration, scope, radicalness, disruptiveness, and number of steps required to implement. |
| G | Design Quality & Packaging | Perceived excellence in how the HPT program is bundled, presented, and assembled. |
| H | Cost | Costs of the HPT program and costs associated with implementing the program including investment, supply, and opportunity (alternative) costs. |
| **II.**  **EXTERNAL CONTEXT** | | This domain is related to the health center’s external environments influencing engagement/implementation success. |
| A | CHC Workforce Needs | The extent to which health center workforce needs, as well as barriers and facilitators to meet those needs, are accurately known and prioritized by the organization. |
| B | Cosmopolitanism | The degree to which an organization is networked with other external organizations. |
| C | External Policy & Incentives | A broad construct that includes external strategies to spread HPT programs, including policy and regulations (governmental or other central entity), external mandates, recommendations and guidelines, pay-for-performance, collaboratives, and public or benchmark reporting. |
| **III. ORGANIZATIONAL CHARACTERISTICS** | | This domain is related to the health center’s internal organizational conditions influencing engagement/implementation success. |
| A | Structural Characteristics | A broad construct that includes the social architecture, age, maturity, and size of an organization, leadership/staff stability, and levels of specialization and integration across the organization. |
| B | Networks & Communications | The nature and quality of webs of social networks and the nature and quality of formal and informal communications within an organization. |
| C | Culture | Norms, values, and basic assumptions of a given organization. |
| D | Implementation Climate | The absorptive capacity for change, shared receptivity of involved individuals to engage with and implement HPT programs, and the extent to which use of training programs will be rewarded, supported, and expected within their organization. |
| 1 | Tension for Change | The degree to which stakeholders perceive the current situation as intolerable or needing change. |
| 2 | Compatibility | The degree of tangible fit between meaning and values attached to the HPT programs by involved individuals and how the intervention fits with existing workflows and systems. |
| 3 | Relative Priority | Individuals’ shared perception of the importance of implementing HPT programs within the organization. |
| 4 | Organizational Incentives & Rewards | Financial incentives (performance reviews and promotions) and less tangible incentives such as increased stature or respect. |
| 5 | Goals and Feedback | The degree to which goals are clearly communicated, acted upon, and fed back to staff, and alignment of that feedback with goals. |
| 6 | Learning Climate | A climate in which: a) leaders express their own fallibility and need for team members’ assistance and input; b) team members feel that they are essential, valued, and knowledgeable partners in the change process; c) individuals feel psychologically safe to try new methods; and d) there is sufficient time and space for reflective thinking and evaluation. |
| E | Readiness to Engage | Tangible and immediate indicators of organizational commitment to its decision *to engage* with an HPT program. |
| F | Readiness to Teach | Tangible and immediate indicators of organizational commitment to its decision *to teach* an HPT program.  The organization has the size, reputation and structures (i.e., recognition as experts) to act as a teaching organization. |
| G | Leadership Engagement | Commitment, involvement, and accountability of leaders and managers to engage with/teach HPT programs. |
| H | Available Resources | The level of resources dedicated for implementation and on-going operations, including money, training, education, physical space, and time. |
| I | Access to Knowledge & Information | Ease of access to digestible information and knowledge about an HPT program and how to incorporate it into the day to day functioning of the organization. |
| **IV.**  **CHARACTERISTICS OF INDIVIDUALS** | | The fourth major domain is about the individuals involved with the HPT implementation process. Individuals can influence this process through their knowledge, attitudes, and behaviors. |
| A | Knowledge & Beliefs about the HPT programs | Attitudes toward and value placed on the HPT programs as well as familiarity with facts, truths, and principles related to the HPT programs. |
| B | Change Efficacy | Shared belief in the conjoint capabilities to implement an HPT program successfully |
| C | Stage of Change | Characterization of the phase an organization is in, as it progresses toward skilled, enthusiastic, and sustained use of an HPT program. |
| D | Individual Identification with Organization | A broad construct related to how individuals perceive the organization, and their relationship and degree of commitment with that organization. |
| E | Other Drivers of Organizational-level Behavior | A broad construct to include concepts from organizational psychology related to implementation of organizational interventions (tolerance of ambiguity, motivation, values, competence, and capacity). |
| **V. PROCESS** | | The fifth domain is associated with the implementation process. This domain is based on the observation that successful engagement usually requires an active change process aimed to achieve effective implementation of the HPT program(s). |
| A | Planning | The degree to which a scheme or method of behavior and tasks for implementing an HPT program are developed in advance, and the quality of those schemes or methods. |
| B | Engaging | Attracting and involving appropriate individuals in the implementation and use of the HPT programs through a combined strategy of social marketing, education, role modeling, training, and other similar activities. |
| 1 | Opinion Leaders | Individuals in an organization who have formal or informal influence on the attitudes and beliefs of their colleagues with respect to implementing the HPT program. |
| 2 | Formally Appointed Internal Implementation Leaders | Individuals from within the organization who have been formally appointed with responsibility for implementing HPT programs as coordinator, project manager, team leader, or other similar roles. |
| 3 | Champions | Individuals who are effective in overcoming indifference or resistance that the implementation of an HPT program may provoke in the organization. |
| 4 | External Change Agents | Individuals who are affiliated with an outside entity who formally influence or facilitate implementation decisions in a desirable direction. |
| C | Executing | Carrying out or accomplishing the implementation according to plan. |
| D | Reflecting & Evaluating | Quantitative and qualitative feedback about the progress and quality of implementation accompanied with regular personal and team debriefing about progress and experience. |

*^a^The constructs and their descriptions are adapted from the Consolidated Framework for Implementation Research (Damschroder et al., 2009)*
