# Supplementary material for: Development and validation of the Readiness to Train Assessment Tool (RTAT)
Source: BMC Health Serv Res. 2021 Apr 28;21:396. doi: 10.1186/s12913-021-06406-3 (PMC8082650; doi:10.1186/s12913-021-06406-3)
Supplement: Supplementary file 2 — Additional file 2. Survey items that were eliminated after factor analysis. [file 12913_2021_6406_MOESM2_ESM.docx]

**Survey items that were eliminated after factor analysis.**

| **Survey Item** |
| --- |
|  |
| The program will be carried out or accomplished according to an implementation plan that... - is realistic and feasible. |
| The program will be carried out or accomplished according to an implementation plan that... - has been developed by the appropriate stakeholders (involved in and actively engaged in the planning process). |
| For this health professions training program, our health center has the reputation and structures to develop and implement instructional content, teaching strategies, and assessments. |
| The infrastructure of our health center (social structure, age, maturity, size, or physical layout) supports the implementation of health professions training. |
| At our health center: Innovation is valued. |
| At our health center: Training the next generation of workforce is an organizational goal. |
| Regarding health professions training, our health center has a strong commitment to act as a teaching organization. |
| Our health center is able to offer appropriate resources to our trainees to help them overcome barriers to participating in health professions training. |
| The supporting evidence for this health professions training program is well received by influential stakeholders (e.g., subject experts, leaders) at our health center. |
| In regard to implementing a specific health professions training program, the leaders and managers at our health center have positive working relationships with the current/proposed implementation team. |
| One or more champions at our health center are enthusiastically pushing for this health professions training program. |
| Our health center has a plan to acquire or develop the resources that are currently NOT available for health professions training program implementation. |
| Our health center is capable of trialing health professions training programs on a small scale and, if necessary, capable of reversing changes. |
| Our health center is willing and able to support the costs (staffing, financial, infrastructure, and opportunity costs) associated with this health professions training program either through organizational or external funding. |
| Regarding health professions training, our health center has clinical experts that are well-poised to serve as instructors, preceptors, and mentors. |
| The following resources are available and sufficient to implement and carry out the health professions training program: - Facilities (e.g., teaching or exam room). |
| The following resources are available and sufficient to implement and carry out the health professions training program: - Information technology (e.g., computers, software, and systems). |
| The following resources are available and sufficient to implement and carry out the health professions training program: - A sufficient number and type of patients. |
| In regard to implementing a specific health professions training program, the leaders and managers at our health center have the authority to carry out the implementation. |
| Our health center is prepared to deal with changes to job requirements and staffing related to health professions training programs. |
| Our health center is prepared to adopt new policies or revise existing organizational policies before implementing any health professions training program. |
| Our health center understands the barriers to the successful implementation of health professions training programs (e.g., costs, time, and attitudes). |
| The majority of staff members at my health center feel that: The program will negatively impact staff productivity due to teaching responsibilities. |
| For this health professions training program, our health center has the reputation and structures to determine the program objectives and the sequence in which to address them. |
